# Supplementary material for: copLAB gene prevalence and diversity among Trinidadian Xanthomonas spp. black-rot lesion isolates with variable copper resistance profiles
Source: PeerJ. 2023 Jun 27;11:e15657. doi: 10.7717/peerj.15657 (PMC10312155; doi:10.7717/peerj.15657)
Supplement: Supplemental Information 1 [file peerj-11-15657-s001.docx]

## Supplemental Table 1. *copLAB* gene locus co-ordinates of local Xsp genomes characterised by Ramnarine, Jayaraman and Ramsubhag (2022).

| Species | Strain | Contig | Gene | Start | Stop |
| --- | --- | --- | --- | --- | --- |
| Xcc | BrA1 | 37 | copB | 29042 | 28005 |
| Xcc | BrA1 | 37 | copA | 30885 | 29047 |
| Xcc | BrA1 | 37 | copL | 31705 | 32118 |
| Xcc | Cf3C | 28 | copB | 26194 | 25157 |
| Xcc | Cf3C | 28 | copA | 28037 | 26199 |
| Xcc | Cf3C | 28 | copL | 28143 | 28568 |
| Xcc | Cf4B1 | 25 | copL | 51024 | 51449 |
| Xcc | Cf4B1 | 25 | copA | 51555 | 53393 |
| Xcc | Cf4B1 | 25 | copB | 53398 | 54435 |
| Xmel | CaNP5B | 19 | copL | 19444 | 19755 |
| Xmel | CaNP5B | 19 | copA | 19869 | 21737 |
| Xmel | CaNP5B | 19 | copB | 21734 | 22858 |
| Xmel | CaNP6A | 20 | copB | 57744 | 56620 |
| Xmel | CaNP6A | 20 | copA | 59609 | 57741 |
| Xmel | CaNP6A | 20 | copL | 59967 | 59773 |
| Xmel | DMCX | 9 | copB | 10681 | 11805 |
| Xmel | DMCX | 9 | copA | 11802 | 13670 |
| Xmel | DMCX | 9 | copL | 13784 | 14203 |

## Supplemental Table 2 Lesion associated isolate PCR classification and identification

| Isolate | Location | RT 16 F/R | XCF/R | RST2/3 | Species | BrA1 copL | BrA1 copA | BrA1 copB | Tr copL | Tr copA | Tr copB | *cop* genotype | CuSo4.5H20 | Cu Sensitivity | Blast ID |
| --- | --- | --- | --- | --- | --- | --- | --- | --- | --- | --- | --- | --- | --- | --- | --- |
| Ar1BCA2 | Aranguez | 1 | 1 | 0 | Xcc | 1 | 1 | 1 | 0 | 0 | 0 | Variant | 400 | R | Xanthomonas |
| Cf3C | Aranguez | 1 | 1 | 0 | Xcc | 1 | 1 | 1 | 0 | 0 | 0 | Variant | 600 | R | Xanthomonas |
| Cf4B | Aranguez | 1 | 1 | 0 | Xcc | 1 | 1 | 1 | 0 | 0 | 0 | Variant | - | - | Xanthomonas |
| BrA1 | Aranguez | 1 | 1 | 0 | Xcc | 1 | 1 | 1 | 0 | 0 | 0 | Variant | 300 | R | Xanthomonas |
| Ca3B | Aranguez | 1 | 1 | 0 | Xcc | 1 | 1 | 1 | 0 | 0 | 0 | Variant | 600 | R | Xanthomonas |
| Cf1A | Aranguez | 1 | 1 | 0 | Xcc | 1 | 1 | 1 | 0 | 0 | 0 | Variant | 600 | R | Xanthomonas |
| Cf3A1 | Aranguez | 1 | 1 | 0 | Xcc | 1 | 1 | 1 | 0 | 0 | 0 | Variant | 600 | R | Xanthomonas |
| Cf4B1 | Aranguez | 1 | 1 | 0 | Xcc | 1 | 1 | 1 | 0 | 0 | 0 | Variant | 600 | R | Xanthomonas |
| Cf5A2 | Aranguez | 1 | 1 | 0 | Xcc | 1 | 1 | 1 | 0 | 0 | 0 | Variant | 600 | R | Xanthomonas |
| Cf5B | Aranguez | 1 | 1 | 0 | Xcc | 1 | 1 | 1 | 0 | 0 | 0 | Variant | 600 | R | Xanthomonas |
| Cf6A1 | Aranguez | 1 | 1 | 0 | Xcc | 1 | 1 | 1 | 0 | 0 | 0 | Variant | 600 | R | Xanthomonas |
| AR1PC2 | Aranguez | 1 | 1 | 0 | Xcc | 0 | 0 | 0 | 0 | 0 | 0 | Undetected | 200 | T | Xanthomonas |
| FRL2D3 | Bon Air | 1 | 1 | 0 | Xcc | 0 | 0 | 0 | 1 | 1 | 1 | Traditional | - | - | Xanthomonas |
| FRL2G4 | Bon Air | 1 | 1 | 0 | Xcc | 0 | 0 | 0 | 1 | 1 | 1 | Traditional | - | - | Xanthomonas |
| FRL1C1 | Bon Air | 1 | 0 | 0 | Xsp | 0 | 0 | 0 | 1 | 1 | 1 | Traditional | 400 | R | Xanthomonas |
| FRL2G5 | Bon Air | 1 | 0 | 0 | Xsp | 0 | 0 | 0 | 0 | 1 | 1 | Traditional | 600 | R | Xanthomonas |
| DMCE | Maloney | 1 | 0 | 0 | Xmel | 0 | 0 | 0 | 1 | 1 | 1 | Traditional | - | - | Xanthomonas melonis |
| DMCA | Maloney | 1 | 0 | 0 | Xmel | 0 | 0 | 0 | 1 | 1 | 1 | Traditional | 600 | R | Xanthomonas melonis |
| DMCK* | Maloney | 1 | 0 | 0 | Xmel | 0 | 0 | 0 | 0 | 0 | 0 | Undetected | 600 | R | Xanthomonas melonis |
| DMCX | Maloney | 1 | 0 | 0 | Xmel | 0 | 0 | 0 | 1 | 1 | 1 | Traditional | 600 | R | Xanthomonas melonis |
| CCB4 | Maloney | 1 | 0 | 1 | Xsp | 0 | 0 | 0 | 1 | 1 | 1 | Traditional | - | - | Xanthomonas |
| CNP2C | Navet | 1 | 1 | 0 | Xcc | 0 | 0 | 0 | 0 | 0 | 0 | Undetected | - | - | Xanthomonas |
| CaNP6B | Navet | 1 | 1 | 0 | Xcc | 0 | 0 | 0 | 1 | 1 | 1 | Traditional | 500 | R | Xanthomonas |
| CNP1E | Navet | 1 | 1 | 0 | Xcc | 0 | 0 | 0 | 1 | 1 | 1 | Traditional | 300 | R | Xanthomonas |
| CNP2A | Navet | 1 | 1 | 0 | Xcc | 0 | 0 | 0 | 0 | 0 | 0 | Undetected | 400 | R | Xanthomonas |
| CNP3C | Navet | 1 | 1 | 0 | Xcc | 0 | 0 | 0 | 0 | 0 | 0 | Undetected | 400 | R | Xanthomonas |
| CaNP1C | Navet | 1 | 1 | 0 | Xcc | 0 | 0 | 0 | 0 | 0 | 0 | Undetected | 100 | S | Xanthomonas |
| CNP4A | Navet | 1 | 1 | 0 | Xcc | 0 | 0 | 0 | 0 | 0 | 0 | Undetected | 200 | T | Xanthomonas |
| CaNP5B | Navet | 1 | 0 | 0 | Xmel | 0 | 0 | 0 | 1 | 1 | 1 | Traditional | 600 | R | Xanthomonas melonis |
| CaNP6A | Navet | 1 | 0 | 0 | Xmel | 0 | 0 | 0 | 1 | 1 | 1 | Traditional | 600 | R | Xanthomonas melonis |
| CaNP1D | Navet | 1 | 0 | 0 | Xmel | 0 | 0 | 0 | 0 | 0 | 0 | Undetected | 200 | T | Xanthomonas melonis |
| PNP44 | Navet | 1 | 0 | 1 | Xsp | 0 | 0 | 0 | 1 | 1 | 1 | Traditional | 600 | R | ND |
| PNP63 | Navet | 1 | 0 | 1 | Xsp | 0 | 0 | 0 | 1 | 1 | 1 | Traditional | 600 | R | ND |
| PNP64 | Navet | 1 | 0 | 1 | Xsp | 0 | 0 | 0 | 1 | 1 | 1 | Traditional | 600 | R | ND |
| PNS3 | Navet | 1 | 0 | 1 | Xsp | 0 | 0 | 0 | 0 | 0 | 0 | Undetected | 600 | R | ND |
| PNP25 | Navet | 1 | 0 | 1 | Xsp | 0 | 0 | 0 | 1 | 1 | 1 | Traditional | 600 | R | Xanthomonas |
| PNP26 | Navet | 1 | 0 | 1 | Xsp | 0 | 0 | 0 | 1 | 1 | 1 | Traditional | 600 | R | Xanthomonas |
| PNP34 | Navet | 1 | 0 | 1 | Xsp | 0 | 0 | 0 | 1 | 1 | 1 | Traditional | 600 | R | Xanthomonas |
| PNP49 | Navet | 1 | 0 | 1 | Xsp | 0 | 0 | 0 | 1 | 1 | 1 | Traditional | 600 | R | Xanthomonas |
| PNP54 | Navet | 1 | 0 | 1 | Xsp | 0 | 0 | 0 | 1 | 1 | 1 | Traditional | 600 | R | Xanthomonas |
| PNP58 | Navet | 1 | 0 | 1 | Xsp | 0 | 0 | 0 | 1 | 1 | 1 | Traditional | 600 | R | Xanthomonas |
| PNP62 | Navet | 1 | 0 | 1 | Xsp | 0 | 0 | 0 | 1 | 1 | 1 | Traditional | 600 | R | Xanthomonas |
| CaNP3B | Navet | 1 | 0 | 0 | Xsp | 0 | 0 | 0 | 1 | 1 | 1 | Traditional | 600 | R | Xanthomonas |
| PNP39 | Navet | 1 | 0 | 1 | Xsp | 0 | 0 | 0 | 1 | 1 | 1 | Traditional | 600 | R | ND |
| PNP72 | Navet | 1 | 0 | 1 | Xsp | 0 | 0 | 0 | 1 | 1 | 1 | Traditional | 600 | R | ND |

## Supplemental Table 3. Environmental population copper sensitivity profiling

| Isolate | Cu Sensitivity | Environment | Field | ID | Genus |
| --- | --- | --- | --- | --- | --- |
| ArS1A1A | R | Soil | Cauliflower | Achromobacter sp. | Achromobacter |
| ArP1B7 | R | Phylloplane | Cauliflower | Pseudomonas aeruginosa | Pseudomonas |
| ArS1A26 | R | Soil | Cauliflower | Pseudomonas monteilli | Pseudomonas |
| ArS1B14a | R | Soil | Cauliflower | Serratia marcescens | Serratia |
| ArP2A12 | R | Phylloplane | Abandoned plot | Sphingomonas sp. | Sphingomonas |
| ArP1B10 | R | Phylloplane | Cauliflower | Stenotrophomonas maltophilia | Stenotrophomonas |
| ArP1B18 | R | Phylloplane | Cauliflower | Stenotrophomonas maltophilia | Stenotrophomonas |
| ArS1B21 | R | Soil | Cauliflower | Stenotrophomonas maltophilia | Stenotrophomonas |
| ArP1A27 | R | Phylloplane | Cauliflower | ND | ND |
| ArP1B26 | R | Phylloplane | Cauliflower | ND | ND |
| ArP1B53 | R | Phylloplane | Cauliflower | Pseudomonas sp. | Pseudomonas |
| ArS1A22 | R | Soil | Cauliflower | ND | ND |
| ArS2A18 | R | Soil | Abandoned plot | Klebsiella pneumoniae | Klebsiella |
| ArS2A33 | R | Soil | Abandoned plot | ND | ND |
| ArP1B23 | R | Phylloplane | Cauliflower | Bacillus cereus | Bacillus |
| ArP1B36 | R | Phylloplane | Cauliflower | Bacillus | Bacillus |
| ArP1B34 | R | Phylloplane | Cauliflower | Stenotrophomonas maltophilia | Stenotrophomonas |
| ArP1A1 | R | Phylloplane | Cauliflower | Acinetobacter sp. | Acinetobacter |
| ArP1A2 | R | Phylloplane | Cauliflower | Acinetobacter sp. | Acinetobacter |
| ArP1A28A | R | Phylloplane | Cauliflower | ND | ND |
| ArP1A4 | R | Phylloplane | Cauliflower | Acinetobacter sp. | Acinetobacter |
| ArP1B35 | R | Phylloplane | Cauliflower | Bacillus | Bacillus |
| ArS1A21 | R | Soil | Cauliflower | Achromobacter sp. | Achromobacter |
| ArS1A23 | R | Soil | Cauliflower | Achromobacter sp. | Achromobacter |
| ArP1A3 | R | Phylloplane | Cauliflower | Acinetobacter calcoaceticus | Acinetobacter |
| ArS1A14 | R | Soil | Cauliflower | Acinetobacter calcoaceticus | Acinetobacter |
| ArS1A19 | R | Soil | Cauliflower | Acinetobacter calcoaceticus | Acinetobacter |
| ArS1A16 | R | Soil | Cauliflower | Acinetobacter sp. | Acinetobacter |
| ArP1B47 | R | Phylloplane | Cauliflower | Bacillus cereus | Bacillus |
| ArP1B8 | R | Phylloplane | Cauliflower | Bacillus cereus | Bacillus |
| ArP2A2 | R | Phylloplane | Abandoned plot | Bacillus cereus | Bacillus |
| ArP2A22 | R | Phylloplane | Abandoned plot | Bacillus cereus | Bacillus |
| ArP2B2 | R | Phylloplane | Abandoned plot | Bacillus cereus | Bacillus |
| ArS2A10 | R | Soil | Abandoned plot | Enterobacter sp. | Enterobacter |
| ArS2A11 | R | Soil | Abandoned plot | Enterobacter sp. | Enterobacter |
| ArS2A31 | R | Soil | Abandoned plot | Enterobacter sp. | Enterobacter |
| ArS2A36 | R | Soil | Abandoned plot | Enterobacter sp. | Enterobacter |
| ArS2A9 | R | Soil | Abandoned plot | Enterobacter sp. | Enterobacter |
| ArS2B2 | R | Soil | Abandoned plot | Enterobacter sp. | Enterobacter |
| ArS2B3 | R | Soil | Abandoned plot | Enterobacter sp. | Enterobacter |
| ArS2A12 | R | Soil | Abandoned plot | Klebsiella pneumoniae | Klebsiella |
| ArS2A14 | R | Soil | Abandoned plot | Klebsiella pneumoniae | Klebsiella |
| ArP1B38 | R | Phylloplane | Cauliflower | Pseudomonas aeruginosa | Pseudomonas |
| ArP1B39 | R | Phylloplane | Cauliflower | Pseudomonas aeruginosa | Pseudomonas |
| ArP1A26 | R | Phylloplane | Cauliflower | Pseudomonas monteilli | Pseudomonas |
| ArP1B2 | R | Phylloplane | Cauliflower | Pseudomonas monteilli | Pseudomonas |
| ArP1B22 | R | Phylloplane | Cauliflower | Pseudomonas monteilli | Pseudomonas |
| ArS1A15 | R | Soil | Cauliflower | Pseudomonas monteilli | Pseudomonas |
| ArS1A24 | R | Soil | Cauliflower | Pseudomonas monteilli | Pseudomonas |
| ArS1A28 | R | Soil | Cauliflower | Pseudomonas monteilli | Pseudomonas |
| ArS2A25 | R | Soil | Abandoned plot | Pseudomonas monteilli | Pseudomonas |
| ArS2A28 | R | Soil | Abandoned plot | Pseudomonas monteilli | Pseudomonas |
| ArS2A29 | R | Soil | Abandoned plot | Pseudomonas monteilli | Pseudomonas |
| ArS2A30 | R | Soil | Abandoned plot | Pseudomonas monteilli | Pseudomonas |
| ArP1A25 | R | Phylloplane | Cauliflower | Pseudomonas sp. | Pseudomonas |
| ArP1B57 | R | Phylloplane | Cauliflower | Pseudomonas sp. | Pseudomonas |
| ArP1B6 | R | Phylloplane | Cauliflower | Serratia marcescens | Serratia |
| ArS1B19 | R | Soil | Cauliflower | Serratia marcescens | Serratia |
| ArS1B16 | R | Soil | Cauliflower | Serratia sp. | Serratia |
| ArS1B14 | R | Soil | Cauliflower | Shigella sp. | Shigella |
| ArS1A12 | R | Soil | Cauliflower | Stenotrophomonas maltophilia | Stenotrophomonas |
| ArS1B11 | R | Soil | Cauliflower | Stenotrophomonas maltophilia | Stenotrophomonas |
| ArS1B17 | R | Soil | Cauliflower | Stenotrophomonas maltophilia | Stenotrophomonas |
| ArS1B18 | R | Soil | Cauliflower | Stenotrophomonas maltophilia | Stenotrophomonas |
| ArS1B19A | R | Soil | Cauliflower | Stenotrophomonas maltophilia | Stenotrophomonas |
| ArS2A7 | R | Soil | Abandoned plot | Stenotrophomonas maltophilia | Stenotrophomonas |
| ArS2A8 | R | Soil | Abandoned plot | Stenotrophomonas maltophilia | Stenotrophomonas |
| ArP1A19 | R | Phylloplane | Cauliflower | ND | ND |
| ArP1A29 | R | Phylloplane | Cauliflower | ND | ND |
| ArP1B25 | R | Phylloplane | Cauliflower | ND | ND |
| ArS1A27 | R | Soil | Cauliflower | ND | ND |
| ArS1B13 | R | Soil | Cauliflower | ND | ND |
| ArS1B20 | R | Soil | Cauliflower | ND | ND |
| ArS2B13 | R | Soil | Abandoned plot | ND | ND |
| ArP2A11 | S | Phylloplane | Abandoned plot | Not Identified | Not Identified |
| ArP1A11 | S | Phylloplane | Cauliflower | Not Identified | Not Identified |
| ArP2A17 | T | Phylloplane | Abandoned plot | Not Identified | Not Identified |
| ArP1A16 | S | Phylloplane | Cauliflower | Not Identified | Not Identified |
| ArP2A18 | S | Phylloplane | Abandoned plot | Not Identified | Not Identified |
| ArP1A21 | S | Phylloplane | Cauliflower | Not Identified | Not Identified |
| ArP1A24 | S | Phylloplane | Cauliflower | Not Identified | Not Identified |
| ArP1A30 | S | Phylloplane | Cauliflower | Not Identified | Not Identified |
| ArP2A13 | T | Phylloplane | Abandoned plot | Not Identified | Not Identified |
| ArS2A4 | T | Soil | Abandoned plot | Not Identified | Not Identified |
| ArS2A4 | S | Soil | Abandoned plot | Not Identified | Not Identified |
| ArS2A20 | T | Soil | Abandoned plot | Not Identified | Not Identified |
| ArS2B16 | S | Soil | Abandoned plot | Not Identified | Not Identified |
| ArS2A23 | T | Soil | Abandoned plot | Not Identified | Not Identified |
| ArS2A27 | S | Soil | Abandoned plot | Not Identified | Not Identified |
| ArS1B1 | S | Soil | Cauliflower | Not Identified | Not Identified |
| ArS1B3 | S | Soil | Cauliflower | Not Identified | Not Identified |
| ArS1B5 | S | Soil | Cauliflower | Not Identified | Not Identified |
| ArS1B7 | S | Soil | Cauliflower | Not Identified | Not Identified |
| ArS1B9 | T | Soil | Cauliflower | Not Identified | Not Identified |
| ArP1B4 | S | Phylloplane | Cauliflower | Not Identified | Not Identified |
| ArP1B14 | T | Phylloplane | Cauliflower | Not Identified | Not Identified |
| ArP1B31 | S | Phylloplane | Cauliflower | Not Identified | Not Identified |
| ArP1B28 | T | Phylloplane | Cauliflower | Not Identified | Not Identified |
| ArP1B30 | S | Phylloplane | Cauliflower | Not Identified | Not Identified |
| ArS2B5 | S | Soil | Abandoned plot | Not Identified | Not Identified |
| ArS2B7 | S | Soil | Abandoned plot | Not Identified | Not Identified |
| ArS2B9 | S | Soil | Abandoned plot | Not Identified | Not Identified |
| ArS2B11 | S | Soil | Abandoned plot | Not Identified | Not Identified |
| ArS2B14 | T | Soil | Abandoned plot | Not Identified | Not Identified |
| ArP2B1 | T | Phylloplane | Abandoned plot | Not Identified | Not Identified |
| ArP2B3 | T | Phylloplane | Abandoned plot | Not Identified | Not Identified |
| ArP2B7 | T | Phylloplane | Abandoned plot | Not Identified | Not Identified |
| ArP2B10 | T | Phylloplane | Abandoned plot | Not Identified | Not Identified |
| ArP2B14 | S | Phylloplane | Abandoned plot | Not Identified | Not Identified |
| ArP2B16 | T | Phylloplane | Abandoned plot | Not Identified | Not Identified |
| ArP2B18A | S | Phylloplane | Abandoned plot | Not Identified | Not Identified |
| ArP2A7 | S | Phylloplane | Abandoned plot | Not Identified | Not Identified |
| ArP2A5 | T | Phylloplane | Abandoned plot | Not Identified | Not Identified |
| ArP2A3 | T | Phylloplane | Abandoned plot | Not Identified | Not Identified |
| ArP2A1 | S | Phylloplane | Abandoned plot | Not Identified | Not Identified |
| ArP2A10 | T | Phylloplane | Abandoned plot | Not Identified | Not Identified |
| ArP2A14 | T | Phylloplane | Abandoned plot | Not Identified | Not Identified |
| ArP1A13 | S | Phylloplane | Cauliflower | Not Identified | Not Identified |
| ArP1A17 | S | Phylloplane | Cauliflower | Not Identified | Not Identified |
| ArP1A6 | T | Phylloplane | Cauliflower | Not Identified | Not Identified |
| ArP1B46 | T | Phylloplane | Cauliflower | Not Identified | Not Identified |
| ArP1B59 | T | Phylloplane | Cauliflower | Not Identified | Not Identified |
| ArP1B44 | T | Phylloplane | Cauliflower | Not Identified | Not Identified |
| ArP1B19 | T | Phylloplane | Cauliflower | Not Identified | Not Identified |
| ArP1B43 | T | Phylloplane | Cauliflower | Not Identified | Not Identified |
| ArP1B56 | T | Phylloplane | Cauliflower | Not Identified | Not Identified |
| ArP1B42 | T | Phylloplane | Cauliflower | Not Identified | Not Identified |
| ArS1A1 | T | Soil | Cauliflower | Not Identified | Not Identified |
| ArS1A3 | T | Soil | Cauliflower | Not Identified | Not Identified |
| ArS1A5 | T | Soil | Cauliflower | Not Identified | Not Identified |
| ArS1A7A | T | Soil | Cauliflower | Not Identified | Not Identified |
| ArP1B51 | S | Phylloplane | Cauliflower | Not Identified | Not Identified |
| ArS2A22 | S | Soil | Abandoned plot | Not Identified | Not Identified |
| ArP1A10 | T | Phylloplane | Cauliflower | Not Identified | Not Identified |
| ArP1A12 | S | Phylloplane | Cauliflower | Not Identified | Not Identified |
| ArP1A14 | S | Phylloplane | Cauliflower | Not Identified | Not Identified |
| ArP1A31 | S | Phylloplane | Cauliflower | Not Identified | Not Identified |
| ArP1A15 | S | Phylloplane | Cauliflower | Not Identified | Not Identified |
| ArP1A22 | T | Phylloplane | Cauliflower | Not Identified | Not Identified |
| ArP1A18 | S | Phylloplane | Cauliflower | Not Identified | Not Identified |
| ArP1A20 | S | Phylloplane | Cauliflower | Not Identified | Not Identified |
| ArP1A9 | S | Phylloplane | Cauliflower | Not Identified | Not Identified |
| ArP1A8 | S | Phylloplane | Cauliflower | Not Identified | Not Identified |
| ArP1A32 | S | Phylloplane | Cauliflower | Not Identified | Not Identified |
| ArP1A7 | S | Phylloplane | Cauliflower | Not Identified | Not Identified |
| ArP1A5 | S | Phylloplane | Cauliflower | Not Identified | Not Identified |
| ArS2A3 | S | Soil | Abandoned plot | Not Identified | Not Identified |
| ArS2A5 | S | Soil | Abandoned plot | Not Identified | Not Identified |
| ArS2A19 | S | Soil | Abandoned plot | Not Identified | Not Identified |
| ArS2A21 | S | Soil | Abandoned plot | Not Identified | Not Identified |
| ArS2A26 | T | Soil | Abandoned plot | Not Identified | Not Identified |
| ArS2B15 | S | Soil | Abandoned plot | Not Identified | Not Identified |
| ArS2B18 | S | Soil | Abandoned plot | Not Identified | Not Identified |
| ArS2B4 | S | Soil | Abandoned plot | Not Identified | Not Identified |
| ArS2B8 | T | Soil | Abandoned plot | Not Identified | Not Identified |
| ArS2B6 | S | Soil | Abandoned plot | Not Identified | Not Identified |
| ArS2B10 | S | Soil | Abandoned plot | Not Identified | Not Identified |
| ArS2B12 | S | Soil | Abandoned plot | Not Identified | Not Identified |
| ArP2B11 | S | Phylloplane | Abandoned plot | Not Identified | Not Identified |
| ArP2B12 | T | Phylloplane | Abandoned plot | Not Identified | Not Identified |
| ArP2B19 | T | Phylloplane | Abandoned plot | Not Identified | Not Identified |
| ArP2B17 | T | Phylloplane | Abandoned plot | Not Identified | Not Identified |
| ArP2B13 | S | Phylloplane | Abandoned plot | Not Identified | Not Identified |
| ArP2B15 | S | Phylloplane | Abandoned plot | Not Identified | Not Identified |
| ArP2B6 | T | Phylloplane | Abandoned plot | Not Identified | Not Identified |
| ArP2B9 | S | Phylloplane | Abandoned plot | Not Identified | Not Identified |
| ArP2A4 | S | Phylloplane | Abandoned plot | Not Identified | Not Identified |
| ArP2A6 | S | Phylloplane | Abandoned plot | Not Identified | Not Identified |
| ArS1B2 | T | Soil | Cauliflower | Not Identified | Not Identified |
| ArS1B4 | S | Soil | Cauliflower | Not Identified | Not Identified |
| ArS1B6 | S | Soil | Cauliflower | Not Identified | Not Identified |
| ArS1B8 | T | Soil | Cauliflower | Not Identified | Not Identified |
| ArP1B27 | S | Phylloplane | Cauliflower | Not Identified | Not Identified |
| ArP1B29 | S | Phylloplane | Cauliflower | Not Identified | Not Identified |
| ArP1B50 | S | Phylloplane | Cauliflower | Not Identified | Not Identified |
| ArP1B33 | S | Phylloplane | Cauliflower | Not Identified | Not Identified |
| ArP1B3 | T | Phylloplane | Cauliflower | Not Identified | Not Identified |
| ArP1B37 | S | Phylloplane | Cauliflower | Not Identified | Not Identified |
| ArP1B95 | T | Phylloplane | Cauliflower | Not Identified | Not Identified |
| ArP1B20 | T | Phylloplane | Cauliflower | Not Identified | Not Identified |
| ArP1B32 | S | Phylloplane | Cauliflower | Not Identified | Not Identified |
| ArP1B5 | S | Phylloplane | Cauliflower | Not Identified | Not Identified |
| ArP1B9 | S | Phylloplane | Cauliflower | Not Identified | Not Identified |
| ArP1B17 | T | Phylloplane | Cauliflower | Not Identified | Not Identified |
| ArP1B12 | S | Phylloplane | Cauliflower | Not Identified | Not Identified |
| ArP1B21 | S | Phylloplane | Cauliflower | Not Identified | Not Identified |
| ArP1B24 | S | Phylloplane | Cauliflower | Not Identified | Not Identified |
| ArP1B31A | S | Phylloplane | Cauliflower | Not Identified | Not Identified |
| ArS1A2 | S | Soil | Cauliflower | Not Identified | Not Identified |
| ArS1A4 | T | Soil | Cauliflower | Not Identified | Not Identified |
| ArS1A8 | S | Soil | Cauliflower | Not Identified | Not Identified |
| ArS1A7 | S | Soil | Cauliflower | Not Identified | Not Identified |
| ArS1A18 | T | Soil | Cauliflower | Not Identified | Not Identified |
| ArS2A21 | S | Soil | Abandoned plot | Not Identified | Not Identified |
| ArS2A16A | S | Soil | Abandoned plot | Not Identified | Not Identified |
| ArS2A16 | S | Soil | Abandoned plot | Not Identified | Not Identified |
| ArS2A8 | S | Soil | Abandoned plot | Not Identified | Not Identified |
| ArS2A19 | S | Soil | Abandoned plot | Not Identified | Not Identified |
